# Phosphoprotein phosphatase activity positively regulates oligomeric pyrin to trigger inflammasome assembly in phagocytes

**DOI:** 10.1128/mbio.02066-23

**Published:** 2023-10-03

**Authors:** Haleema S. Malik, Flora Magnotti, Nicole A. Loeven, Jose M. Delgado, Arminja N. Kettenbach, Thomas Henry, James B. Bliska

**Affiliations:** 1 Department of Microbiology and Immunology, Geisel School of Medicine at Dartmouth, Hanover, New Hampshire, USA; 2 CIRI, Centre International de Recherche en Infectiologie, Inserm U111, Université Claude Bernard Lyon, CNRS, UMR5308, ENS de Lyon, Univ Lyon, Lyon, France; 3 Department of Pathology and Laboratory Medicine, Hospital of the University of Pennsylvania, Philadelphia, Pennsylvania, USA; 4 Department of Biochemistry and Cell Biology, Geisel School of Medicine at Dartmouth, Hanover, New Hampshire, USA; 5 Dartmouth Cancer Center, Lebanon, New Hampshire, USA; Yale University School of Medicine, New Haven, Connecticut, USA

**Keywords:** phosphoprotein phosphatase, pyrin, inflammasome, phagocyte

## Abstract

Pyrin is a pattern-recognition receptor in phagocytes that triggers caspase-1 inflammasome assembly in response to bacterial toxins and effectors that inactivate RhoA. Pyrin contains oligomerization domains and is negatively regulated by phosphorylation of two residues, S205 and S241 (murine) or S208 and S242 (human), via the kinases PKN1/2, which are activated by RhoA. Familial Mediterranean Fever (FMF) is caused by the phagocyte production of pyrin gain of function variants, which have a lower threshold for inflammasome assembly upon RhoA-PKN axis inhibition. Inactivation of the RhoA-PKN axis removes negative regulation but a phosphoprotein phosphatase (PPP) is needed to positively regulate pyrin. No PPP that dephosphorylates pyrin has been identified, oligomerization of murine pyrin has not been studied, and the phosphorylation status of oligomeric pyrin is unknown. We used murine macrophages and FMF patient’s monocytes combined with the use of bacterial agonists and chemical inhibitors, native PAGE, phospho-specific antibodies, and siRNA knockdowns to determine if a PPP positively regulates oligomeric pyrin. Results with broadly specific inhibitors indicate that PPP activity is required to dephosphorylate murine and human pyrin in wild-type or FMF patient’s phagocytes. Findings from native PAGE show that murine pyrin is oligomeric and phosphorylated on S205 prior to RhoA inactivation. PPP inhibitors cause reduced mobility of murine pyrin on native PAGE and increased phosphorylation of S242 in human pyrin, suggesting a PPP constitutively counterbalances PKN to regulate second-site phosphorylation. Data from siRNA knockdown experiments implicate PP2A in dephosphorylation of S205 and positive regulation of pyrin in response to RhoA inactivation.

**IMPORTANCE:**

Pyrin, a unique cytosolic receptor, initiates inflammatory responses against RhoA-inactivating bacterial toxins and effectors like *Yersinia’s* YopE and YopT. Understanding pyrin regulation is crucial due to its association with dysregulated inflammatory responses, including Familial Mediterranean Fever (FMF), linked to pyrin gene mutations. FMF mutations historically acted as a defense mechanism against plague. Negative regulation of pyrin through PKN phosphorylation is well established, with *Yersinia* using the YopM effector to promote pyrin phosphorylation and counteract its activity. This study highlights the importance of phosphoprotein phosphatase activity in positively regulating pyrin inflammasome assembly in phagocytic cells of humans and mice. Oligomeric murine pyrin has S205 phosphorylated before inflammasome assembly, and this study implicates the dephosphorylation of murine pyrin S205 by two catalytic subunits of PP2A in macrophages. These findings offer insights for investigating the regulation of oligomeric pyrin and the balance of kinase and phosphatase activity in pyrin-associated infectious and autoinflammatory diseases.

## INTRODUCTION

Numerous Gram-negative bacterial pathogens encode type III secretion systems (T3SSs) that are essential for virulence (1). T3SSs translocate effectors into or across the plasma membrane and into the eukaryotic cytosol during bacterial-host cell contact. Translocated effectors can inhibit innate immune responses to promote pathogenesis (1, 2). Host cells can sense perturbations caused by T3SSs and/or effectors and produce compensatory innate immune responses to counteract infection by pathogens (3, 4). Perturbations induced by T3SSs and/or effectors in macrophages infected with bacterial pathogens can result in the assembly of caspase-1 inflammasomes (5, 6). Inflammasomes are multimeric complexes that are assembled in response to various danger signals in the cell cytosol and serve as a molecular platform for the recruitment and maturation of caspase-1 into its active form (5, 6). Active caspase-1 cleaves gasdermin-D (GSDMD), pro-IL-1β, and pro-IL-18 (7 – 9). The N-terminal domain of GSDMD forms pores in the plasma membrane which allow for the release of mature IL-1β and IL-18 (7 – 9). GSDMD pores can also promote cell death, termed pyroptosis, resulting in the release of additional proinflammatory molecules (10). Pyroptosis amplifies the immune response by restricting the growth of bacteria and facilitating bacterial clearance by neutrophils (7). Virulent bacterial pathogens can inhibit caspase-1 inflammasomes in host cells to counteract protective immune responses mediated by IL-1β, IL-18, and pyroptosis (11).

*Yersinia* species are Gram-negative bacterial pathogens that cause invasive human infections ranging in severity from plague (*Y. pestis*) to mesenteric lymphadenitis (*Y. pseudotuberculosis, Yptb*). These bacteria replicate in lymphoid tissues as extracellular microcolonies and require a plasmid-encoded T3SS for virulence (12). The T3SS delivers seven effectors called *Yersinia* outer proteins (Yops) into the cytosol of phagocytes that are in contact with *Yersinia* (12). The effectors inhibit key host cell processes, including phagocytosis and proinflammatory cytokine production (13). For example, YopE and YopT inhibit phagocytosis by inactivating Rho GTPases, including RhoA (13). YopE mimics a Rho GTPase-activating protein (GAP) to accelerate GTP hydrolysis to GDP in RhoA (14), while YopT is a cysteine protease that cleaves RhoA at its C-terminus so that it is released from the membrane (15). Two effectors that are essential virulence factors inhibit inflammasome activation: YopM, a leucine-rich repeat (LRR)-containing protein (16 – 18), and YopK (19, 20). YopM inhibits the pyrin inflammasome (17, 18). YopK interacts with *Yersinia* translocon components to limit the activation of NLRP3 and NLRC4 inflammasomes (19).

Inactivation of RhoA by YopE or YopT in macrophages triggers the assembly of the pyrin inflammasome as an example of effector-triggered immunity (17, 18, 21, 22). Pyrin is a cytosolic pattern recognition receptor (PRR) that specifically senses pathogen-induced modifications to RhoA (or RhoB or RhoC) to assemble an inflammasome (23). RhoA, RhoB, and RhoC appear to be functionally redundant in regulating pyrin activity (23) and will be referred to as RhoA from here on. Inactivation of RhoA by the TcdB toxin of *Clostridioides difficile* or the TecA effector of *Burkholderia cenocepacia* also triggers pyrin inflammasome assembly in macrophages (23, 24). Pyrin is encoded by the *MEFV* gene (*Mefv* in mice) whose expression is restricted to phagocytes such as neutrophils, monocytes, and activated macrophages (25). Structurally, murine [808 amino acids (isoform 1)] and human (781 amino acids) pyrin are similar except for their C-terminus. Both contain an N-terminal pyrin domain (PYD), followed by a linker region, a B-box, and coiled-coil (CC) domains. Murine pyrin lacks the B30.2 domain present in the C-terminus of human pyrin, and instead, it has a short undefined C-terminal tail (21, 26). Recent evidence shows that the B30.2 domain negatively regulates pyrin inflammasome assembly (27). Pyrin is a TRIM protein, and like other members of this family (28), human pyrin forms dimers via coiled coil (CC) domains (29). Additionally, human pyrin has been shown to form trimers (30). Pyrin potentially forms higher order oligomers (i.e., dimers of dimers), via B-box domains (21). It is not known if the murine pyrin forms oligomers. Despite the difference in structure at the C-terminus of human and murine pyrin, the mechanism of pyrin regulation is similar in both species (31).

Negative regulation of pyrin occurs in a manner similar to the guard hypothesis observed in plants, whereby a plant disease resistance PRR recognizes modifications to host proteins made by pathogens and induces an innate response (32). More specifically, pyrin follows an indirect guard hypothesis in which it does not directly interact with but is negatively regulated by RhoA (23). Hence, pyrin acts as the guard of RhoA (guardee) to protect host cells from bacterial effectors or toxins that target it. RhoA functions through a switch-like mechanism between an “on” (Rho-GTP) and “off” (Rho-GDP) state (Fig. S1). RhoA has numerous roles in phagocytosis, cell cycle, and migration and is targeted by multiple bacterial pathogens (23, 33). Pyrin is negatively regulated by serine phosphorylation, via the kinase PKN (also known as PRK), which in turn is activated by RhoA (Fig. S1). PKN phosphorylates murine pyrin on serines 205 and 241 (S208 and S242 in human pyrin) within 14-3-3-binding sites located in the linker region to negatively regulate pyrin (31, 34). Mutational analysis of S205 and S241 by alanine substitution in ectopically expressed murine pyrin indicates that loss of either site results in partial inflammasome activation, and when both sites are disrupted, there is an additive effect (31). Similar studies of S208 and S242 in human pyrin show that the inflammasome is activated when either site is substituted (34, 35). Moreover, an S242R mutation in *MEFV* causes an autoinflammatory disease with a distinct clinical phenotype, PAAND, which leads to defective 14-3-3 binding and a constitutively active form of pyrin and further underscores the importance of linker phosphorylation in pyrin regulation (36). When pyrin is phosphorylated, the N-terminal pyrin domain (PYD) may be sequestered, preventing the interaction with ASC (21). The B30.2 domain in human pyrin negatively regulates inflammasome assembly downstream of pyrin dephosphorylation and upstream of ASC recruitment (27). It is not known if murine pyrin C-terminal tail also acts in a similar manner to the B30.2 domain. RhoA inactivation removes the negative regulation axis of pyrin, presumably resulting in dephosphorylation of S205/S208 and S241/S242 (21, 22, 31, 34). These findings lead to a model wherein when both serines in the pyrin linker are dephosphorylated, 14-3-3 detaches, the PYD is exposed, and ASC and caspase-1 are recruited to assemble inflammasomes (Fig. S1) (21, 31, 34). A limitation of this model is that no phosphoprotein phosphatase (PPP) that dephosphorylates pyrin has been identified, and the phosphorylation status of pyrin oligomers is unknown.

Assembly of the pyrin inflammasome in phagocytes infected with WT *Yersinia* is blocked due to the action of YopM (17, 18). YopM binds to and activates the protein PKN and, a second kinase, RSK (37). YopM hijacks PKN and RSK to keep pyrin phosphorylated (17, 21, 35). YopM can interact directly with pyrin at its N-terminus and facilitate pyrin’s inactivation by hijacking the RSK and PKN kinases to keep pyrin phosphorylated (35). Virulence of a *Yptb ∆yopM* mutant is restored in *Mefv* ^−/−^ mice, demonstrating that YopM promotes virulence by inhibiting pyrin (17). The importance of pyrin in pathogenesis extends beyond *Yersinia. C. difficile* and *B. cenocepacia,* as well as numerous other bacteria including *Clostridium botulinum*, *Vibrio parahaemolyticus,* and *Histophilus somni,* secrete toxins that trigger the assembly of this inflammasome in phagocytes (23).

Codon changes in the *MEFV* gene (e.g., M694V) that result in the gain of function pyrin variants are responsible for the monogenic human autoinflammatory disease Familial Mediterranean Fever (FMF) (25, 35). FMF is characterized by recurrent episodes of fever and serositis, and it primarily affects eastern Mediterranean populations (38). Magnotti et al. demonstrated that pyrin dephosphorylation was sufficient to promote inflammasome activation using PKN inhibitors in monocytes from FMF patients, which implies that these patients have a lower threshold for pyrin activation than healthy patients (39). It has been hypothesized that the high carrier frequency of FMF in Mediterranean and Middle Eastern populations is the result of a selective advantage in resistance to an unknown infection (35). Population genetic evidence is consistent with the idea that plague selected for FMF mutations in Middle Eastern populations (35). In addition, infection assays with human cells and mice provided evidence that pyrin FMF variants promote resistance to *Y. pestis* (35). These findings underscore the importance of the pyrin inflammasome for human health. Therefore, it is important to understand the dual role of pyrin in promoting immune responses against pathogens and autoinflammation in FMF, and a deeper understanding of pyrin inflammasome regulation will aid in this goal.

Here we used primary murine macrophages, human monocyte cell lines, and primary monocytes from FMF patients in conjunction with bacterial toxins or effectors or chemical inhibitors, native PAGE, immunoblotting with phospho-specific pyrin antibodies, and siRNA knockdowns to determine if PPP activity is required to dephosphorylate oligomeric pyrin and positively regulate inflammasome assembly in response to the inactivation of the Rho-PKN axis.

## RESULTS

### PPP activity is required for pyrin dephosphorylation and positive regulation of inflammasome assembly

**Fig 1:**
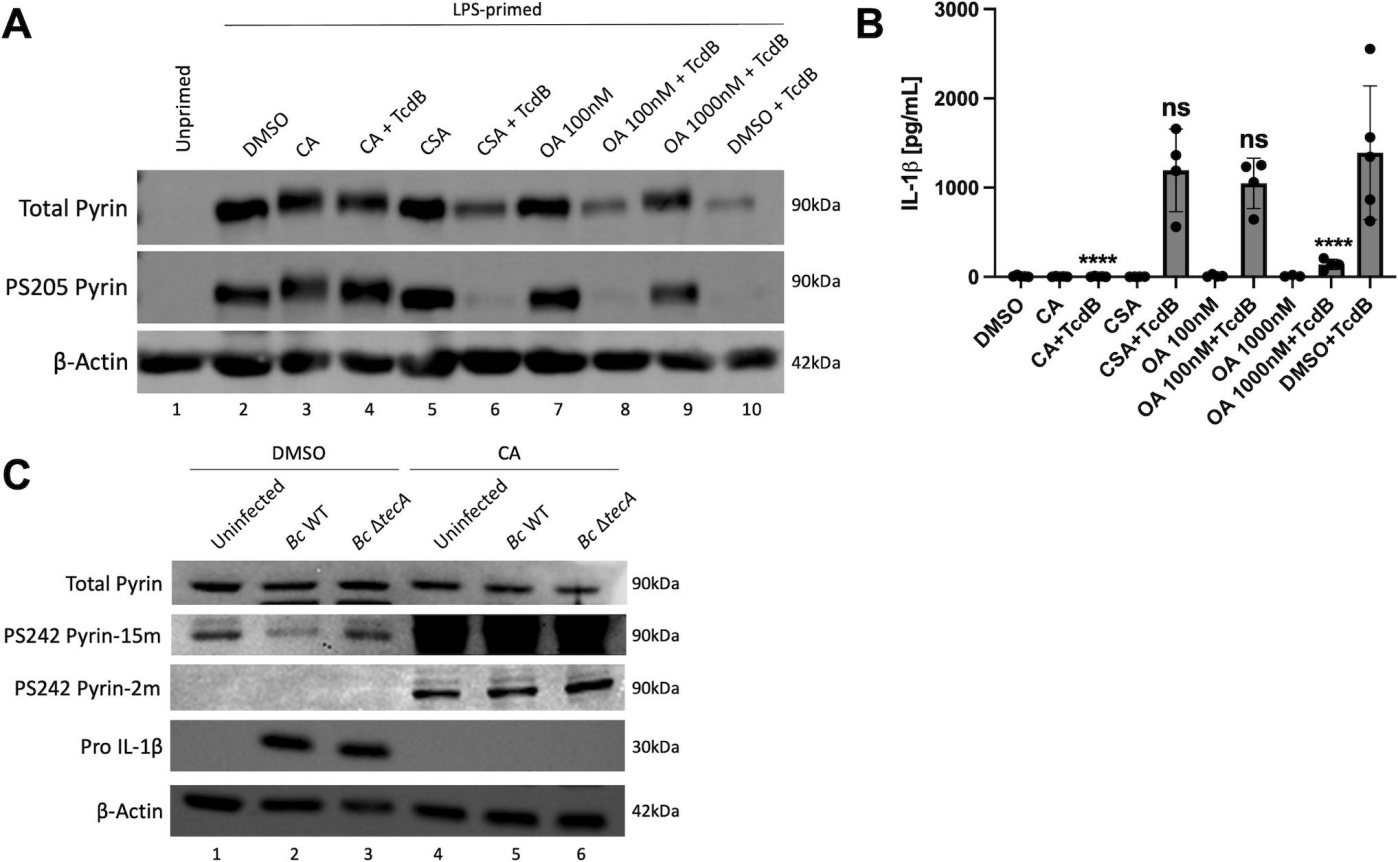
PPP inhibition with CA or OA prevents pyrin dephosphorylation and reduces IL-1β release. LPS-primed BMDMs were pretreated for 15 min and maintained with either 10 nM calyculin A (CA), 100 nM okadaic acid (OA), 1,000 nM OA or 1,000 nM cyclosporin A (CSA), and left unintoxicated or intoxicated with TcdB for 90 min (**A and B**). Unprimed BMDMs or treatment with vehicle DMSO were used as controls. (**A**) Immunoblot analysis of BMDM lysates. (**B**) Mature IL-1β in supernatants as quantified by ELISA. In (**B**), two-way ANOVA with Bonferroni post-hoc correction was applied to calculate significance, and *P*-values as compared to DMSO + TcdB are indicated. *P* < 0.05 was considered significant; *P* < 0.0001 (****); not significant (ns). Each data group is presented as an average (error bars are standard deviation) of at least three independent experiments. Naïve THP-1 cells were pretreated for 15 min and maintained with either DMSO or 10 nM CA, and either left uninfected or infected with *B. cenocepacia* (Bc) WT or Δ*tecA* for 180 min at an MOI of 20 (**C**). Immunoblot analysis of THP-1 lysates is shown. PS242 pyrin signals are shown with long (15 m) or short (2 m) exposures for comparison.

Inactivation of RhoA removes negative regulation by PKN; however, a PPP is needed to positively regulate pyrin by serine dephosphorylation. There are seven members in the PPP family: PP1, PP2A, PP2B (also known as calcineurin or PP3), PP4, PP5, PP6, and PP7 (40, 41). Bone marrow-derived macrophages (BMDMs) were primed with LPS to induce pyrin expression as unprimed cells do not express pyrin (Fig. 1A; Fig. S2A). BMDMs were pretreated for 15 min with DMSO as a vehicle control or a PPP inhibitor to test the role of PPPs in dephosphorylation of pyrin. The following PPP inhibitors were used: calyculin A (CA) at 10 nM or okadaic acid (OA) at 1,000 nM inhibits PP1, PP2A, PP4, PP5, and PP6 but not PP2B; OA at 100 nM more specifically inhibits PP2A; or 1,000 nM of cyclosporin A (CSA) inhibits PP2B. After pretreatment, the BMDMs were maintained in the presence of DMSO or the inhibitors during intoxication for 90 min with *C. difficile* TcdB, which glucosylates and inactivates RhoA (42), to trigger pyrin inflammasome assembly (23). BMDM lysates were analyzed by immunoblotting with monoclonal antibodies specific for total pyrin or phosphoserine 205 (PS205) (31) or actin as loading control. We were unable to monitor PS241 in murine pyrin because the antibody reported to recognize this modification (31) did not work in our hands for this purpose but did recognize human pyrin PS242 (see Fig. 1C). Upon TcdB treatment, pyrin was dephosphorylated at S205, and there was a decrease in soluble total pyrin level (Fig. 1A), which can be due to an increase in pyrin insolubility (22). We found that the treatment of BMDMs with CA or 1,000 nM OA prevented pyrin S205 dephosphorylation in response to TcdB intoxication (Fig. 1A). Results of ELISA on BMDM supernatants showed that CA or 1,000 nM OA also significantly reduced the release of mature IL-1β during TcdB intoxication (Fig. 1B). CSA treatment did not inhibit pyrin S205 dephosphorylation or IL-1β release upon TcdB intoxication (Fig. 1A and B), which indicates that PP2B does not regulate the pyrin inflammasome; 100 nM OA treatment was also ineffective (Fig. 1A and B). We noted that CA and 1,000 nM OA in the absence or presence of TcdB caused an upshift in the positions of pyrin bands on the immunoblots, but not a corresponding increase in PS205 signal (Fig. 1A). This suggests that S205 pyrin is uniformly phosphorylated, and a PPP constitutively dephosphorylates pyrin at an additional site(s), possibly S241, and when this phosphatase is inhibited, hyperphosphorylation (increased phosphorylation) by PKN results in reduced mobility of pyrin by SDS-PAGE. However, it is not known what percentage of pyrin molecules are phosphorylated on S205 and S241. In addition, these results suggest that a PPP other than PP2B is required to dephosphorylate pyrin S205 and trigger the assembly of inflammasome in response to TcdB.

*B. cenocepacia* infection of naive THP-1 cells has been shown to trigger the assembly of the pyrin inflammasome (43). *B. cenocepacia’s* effector protein TecA deamidates RhoA at N41 residue and leads to its inactivation (23, 24). To determine if PPP inhibition prevents dephosphorylation of human pyrin, we infected THP-1 cells with *B. cenocepacia* WT or *∆tecA* strains in the absence or presence of DMSO or CA. Lysates were immunoblotted with antibodies that recognize total pyrin, PS242, pro-IL-1β, or actin as loading control. *B. cenocepacia* infection triggered the dephosphorylation of S242 in a TecA-dependent manner in DMSO-treated THP-1 cells (Fig. 1C). Interestingly, when THP-1 cells were pretreated and maintained in the presence of CA, S242 became hyperphosphorylated in both uninfected and infected conditions (Fig. 1C), which was evident from comparing the short and long exposures of the immunoblots. The hyperphosphorylation of pyrin in the presence of CA in uninfected THP-1 cells suggests that a PPP is constitutively counterbalancing PKN to keep S242 hypophosphorylated (reduced phosphorylation). CA treatment prevented infection-induced production of pro-IL-1β as determined by immunoblotting (Fig. 1C), and as a result, the impact of the inhibitor on the release of mature IL-1β from the THP-1 cells could not be measured. These data indicate that a PPP dephosphorylates pyrin S242 in response to the inactivation of RhoA by TecA in THP-1 cells, but it appears that a PPP is acting constitutively to keep most of the pyrin in the cell dephosphorylated at this site prior to infection.

**Fig 2:**
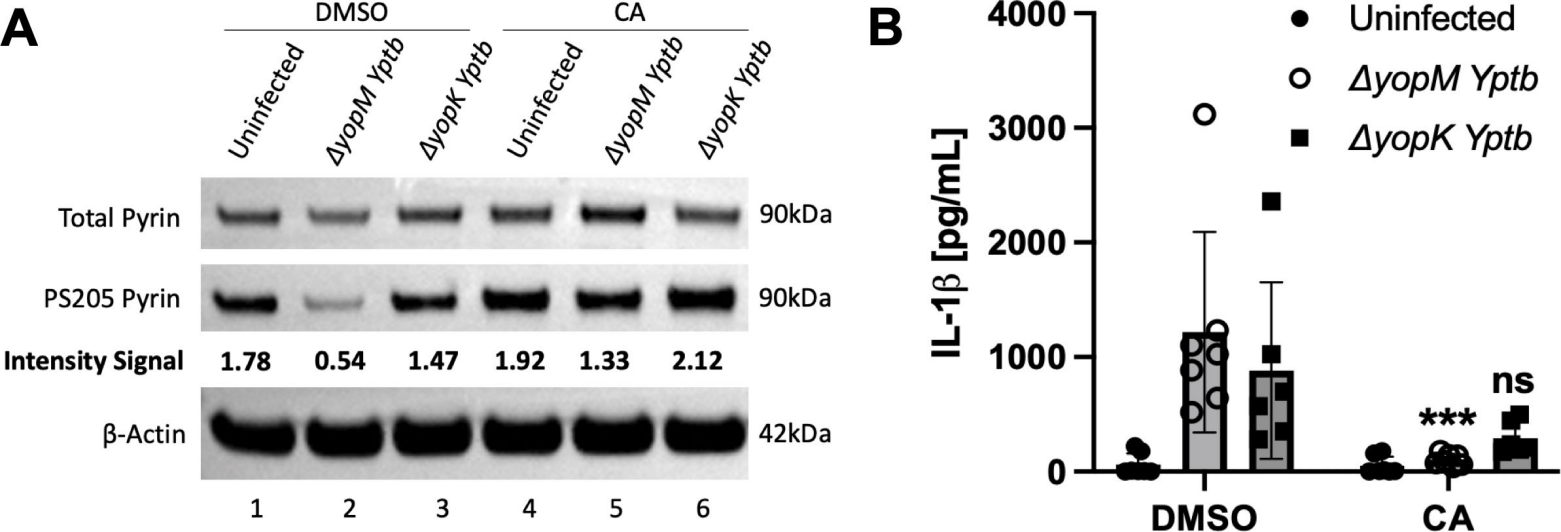
PPP inhibition with CA reduces pyrin dephosphorylation and IL-1β release in BMDMs infected with *∆yopM Yptb*. LPS-primed BMDMs were pretreated for 15 min and maintained with either DMSO or 10 nM CA and infected with the *∆yopM* or *∆yopK* strain at an MOI of 30 for 90 min. Treated uninfected BMDMs were analyzed in parallel. (**A**) Immunoblot analysis. Quantified immunoblot band intensity signals from one experiment representing PS205 pyrin/total pyrin are indicated below the respective blot image. (**B**) Mature IL-1β in supernatants as quantified by ELISA. Two-way ANOVA with Bonferroni post-hoc correction was applied to calculate the significance, and *P*-values of each treatment as compared to its corresponding DMSO-treated infected strain are indicated. *P* < 0.05 was considered significant; *P* < 0.001 (***); not significant (ns). Each data group is presented as an average (error bars are standard deviation) of at least three independent experiments.

We used CA treatment to determine if a PPP is important for YopE and YopT to trigger pyrin S205 dephosphorylation and inflammasome assembly in BMDMs infected with *Yptb*. BMDMs treated with DMSO or CA as above were infected with a *∆yopM* mutant to activate the pyrin inflammasome. We also infected DMSO or CA-treated BMDMs with a *∆yopK* mutant, which triggers the assembly of NLRP3 inflammasome (and to a lesser degree the NLRC4 inflammasome) (19, 20), since there is evidence that PP2A is important for the dephosphorylation and activation of NLRP3 (44). Immunoblot and ELISA analysis of BMDMs pretreated with CA followed by *∆yopM* infection showed that pyrin remained phosphorylated on S205, and there was a significant decrease in IL-1β release (Fig. 2A and B). Results of infection with *∆yopK* also showed reduced IL-1β release when the BMDMs were treated with CA, although the decrease was not significant (Fig. 2B). Pyrin was not dephosphorylated on S205 in response to *∆yopK* infection of untreated BMDMs as expected (Fig. 2A). The finding that CA had a stronger inhibitory effect on IL-1β secretion with *∆yopM* as compared to *∆yopK* infection (Fig. 2B) might reflect residual NLRC4 inflammasome assembly triggered by the latter strain (19).

Similar results were obtained when CA was used to treat BMDMs infected with *∆yopM Yptb* strains expressing only active YopE (with catalytically inactive YopT; *yopT^C139A^*) or YopT (with catalytically inactive YopE; *yopE^R144A^*) (Fig. S2A and B). In addition, immunoblotting indicated that CA did not decrease the steady-state levels of pro-IL-1β (Fig. S2A).

**Fig 3:**
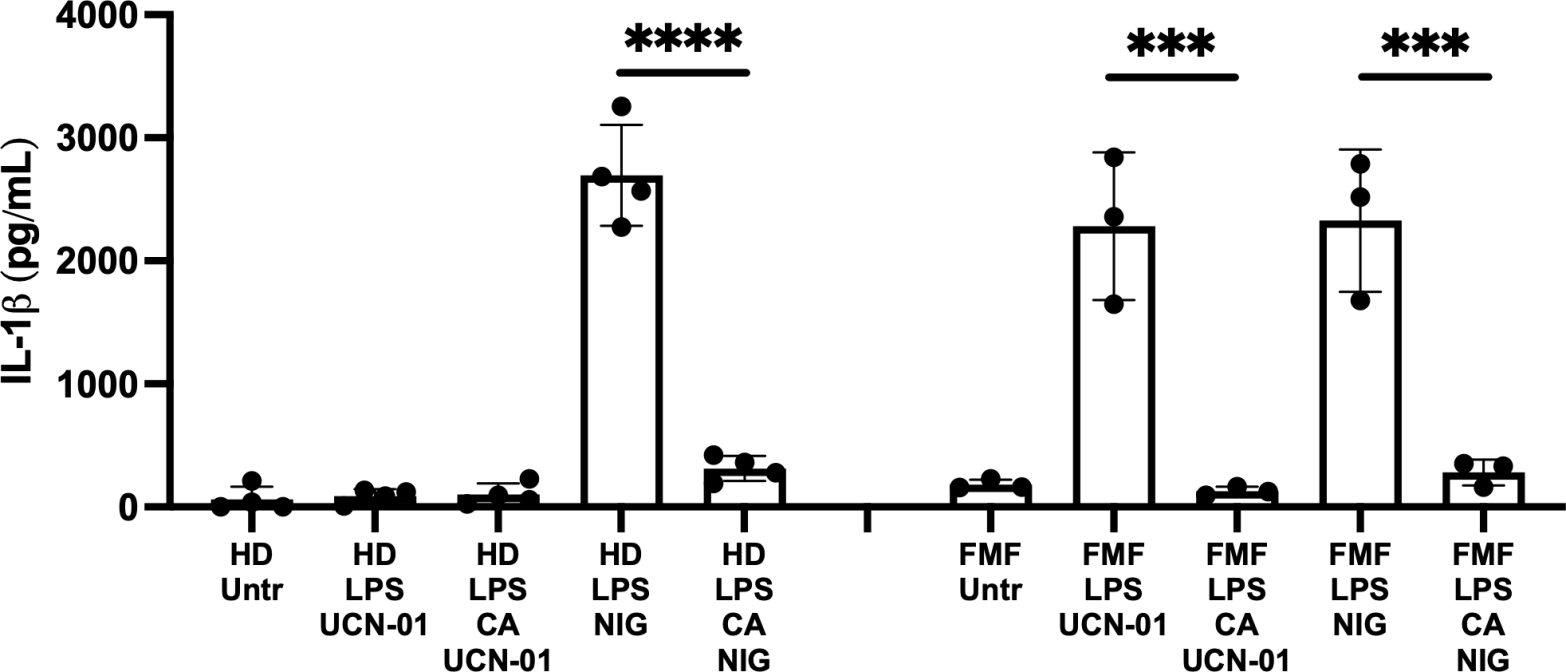
CA inhibits UCN-01 and nigericin-mediated IL-1β release from primary monocytes. Monocytes from four healthy donors (HD) and three FMF patients were primed first with LPS (10 ng/mL) during 2.5 h and, when indicated, pretreated with CA (40 nM) during 30 min. Cells were then treated with 12.5 µM UCN-01 or 5 µg/mL nigericin (NIG) for 90 min. Untreated cells (Untr) were analyzed in parallel. Mature IL-1β in supernatants as quantified by ELISA is shown. Each symbol presents an average (error bars represent standard deviation) of three technical replicates for one patient. Two-way ANOVA with Bonferroni post-hoc correction for multiple comparisons was applied separately to the HD and FMF data. *P* < 0.05 was considered significant; *P* < 0.001 (***) and *P* < 0.0001 (****).

PKN inhibitors activate pyrin inflammasome assembly and pyroptosis in monocytes from FMF patients with the M694V mutation (39). The effect of CA on the release of IL-1β from FMF patient’s monocytes activated by treatment with the PKN inhibitor, UCN-01, was measured. Monocytes from healthy donors were used as a control and as expected did not release IL-1β upon UCN-01 treatment (Fig. 3). Primary monocytes were also treated with nigericin to test the impact of CA on NLRP3 inflammasome activation. Previous studies have shown that using higher concentrations of CA (50 nM) and OA (1 or 2 µM) inhibits IL-1β release in response to various NLRP3 inflammasome agonists (44, 45). In line with these observations, 40 nM CA prevented IL-1β secretion in response to nigericin in primed monocytes from both healthy donor and FMF patients (Fig. 3). Pretreating LPS-primed FMF monocytes with 40 nM CA followed by UCN-01 treatment significantly inhibited IL-1β release as compared to UCN-01 treatment alone (Fig. 3). Moreover, CA treatment also prevented UCN-01 mediated IL-1β release and cell death as measured by propidium iodide influx in a human monocytic cell line, U937, expressing the p.M694V *MEFV* variant (Fig. S3). Thus, in an FMF context where the threshold of activation has been shown to be lower and relies on pyrin dephosphorylation (39, 46), a PPP is constitutively countering the effect of the PKN kinases, and the inhibition of PPP activity prevents inflammasome activation. Altogether these data replicate the inhibitory effect of CA as seen on murine pyrin dephosphorylation and activation in BMDMs and in human cells.

To further delineate which PPP activities positively regulate pyrin, we used tautomycetin (TTN), a more specific inhibitor of PP1 (47, 48). WT BMDMs were pretreated for 15 min and maintained in DMSO or 1 or 2 µM of TTN during infection with *∆yopM Yptb* (Fig. S2C and D). We found that the treatment of BMDMs with TTN did not prevent pyrin PS205 dephosphorylation or IL-1β release in response to infection with *∆yopM Yptb* (Fig. S2C and D). Longer pretreatment of BMDMs with 1 µM TTN also did not decrease IL-1β secretion upon *∆yopM* infection (unpublished observation). Our combined results from the treatment of BMDMs with various phosphatase inhibitors suggest that a PPP (or PPPs) other than PP1 and PP2B is required to activate pyrin.

### PPP activity regulates the phosphorylation of oligomeric pyrin

Although human pyrin has been shown to form trimers (30), the oligomeric status of murine pyrin has not been investigated. In addition, it is not known if pyrin oligomers are differentially phosphorylated or dephosphorylated as compared to monomeric pyrin. We used blue native PAGE (BN-PAGE) and immunoblotting (49, 50) to examine the impact of RhoA inactivation and PPP inhibition on the phosphorylation of oligomeric pyrin. To optimize the procedure, we first tested different nonionic detergent extraction conditions on untreated uninfected WT or *Mefv* ^−/−^ BMDMs. As shown in Fig. S4, when extracts of untreated BMDMs were separated by BN-PAGE and immunoblotted for total (A) or PS205 (B) pyrin, we detected oligomers of pyrin in WT but not *Mefv^−/^* ^−^ BMDMs. Monomeric pyrin (~90 kDa) was not detected, and based on the apparent molecular weight of the oligomers, we tentatively assigned them as dimer (~242 kDa) and higher-order oligomers (~480–720 kDa). Thus, most of the pyrin in BMDMs is in a higher oligomeric form that can be phosphorylated on S205. Recovery of total and PS205 pyrin was optimal using 0.5% or 1% digitonin (Fig. S4), and 1% was used going forward.

**Fig 4:**
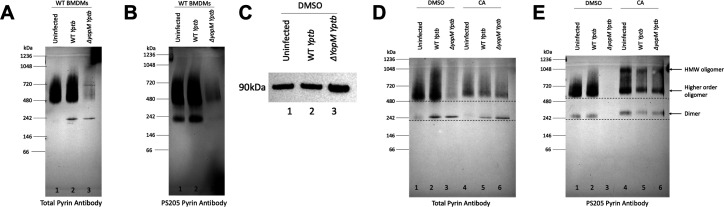
Impact of PPP inhibition with CA and *Yptb* infection on pyrin oligomers and phosphorylation as determined by BN-PAGE and immunoblotting. WT BMDMs left untreated (**A and B**) or pretreated and maintained with DMSO or 10 nM CA (**C–E**) were left uninfected or infected with WT or *∆yopM Yptb* at an MOI of 30 for 90 min. BMDM lysate samples prepared in 1% digitonin were separated by BN-PAGE and immunoblotted. Insoluble proteins from 1% digitonin lysates of DMSO-treated samples were analyzed by SDS-PAGE and total pyrin immunoblotting (**C**). BN-PAGE MW standards are shown on the left, and tentative positions of pyrin oligomers are shown on the right. Dashed horizontal lines in (**D and E**) aid viewer appreciation of band upshifts in the presence of CA.

We next analyzed the phosphorylation and oligomeric status of pyrin in BMDMs infected with WT or *∆yopM Yptb* in the absence of CA using BN-PAGE and immunoblotting. As shown in Fig. 4, the total (A) and PS205 (B) pyrin oligomer signals decreased in BMDMs infected with *∆yopM* (lane 3). Loss of the total protein signal was largely due to increased insolubility of the dephosphorylated form of pyrin when analyzed in the insoluble fraction using nonionic detergent (Fig. 4C) as we have documented previously as well (22). Some degradation of pyrin upon *∆yopM* infection is also possible. This insolubility issue prevented us from determining if the oligomeric state of pyrin is altered upon dephosphorylation. In BMDMs treated with CA, there was an upshift in the positions of the dimeric and higher oligomeric forms under all conditions, most evident with the PS205 signal, and there was no insolubility or dephosphorylation of pyrin in response to infection with *∆yopM* (Fig. 4D and E). In addition, in the presence of CA, a very high molecular weight oligomer at ~1,048 kDa was detected with the PS205 pyrin signal under all conditions (Fig. 4E). These results confirm the upshift in mobility due to hyperphosphorylation of pyrin in the presence of CA as seen in some SDS-PAGE immunoblots (Fig. 1A). These data further suggest that hyperphosphorylation of pyrin increases oligomerization.

### YopM interacts with oligomeric pyrin

**Fig 5:**
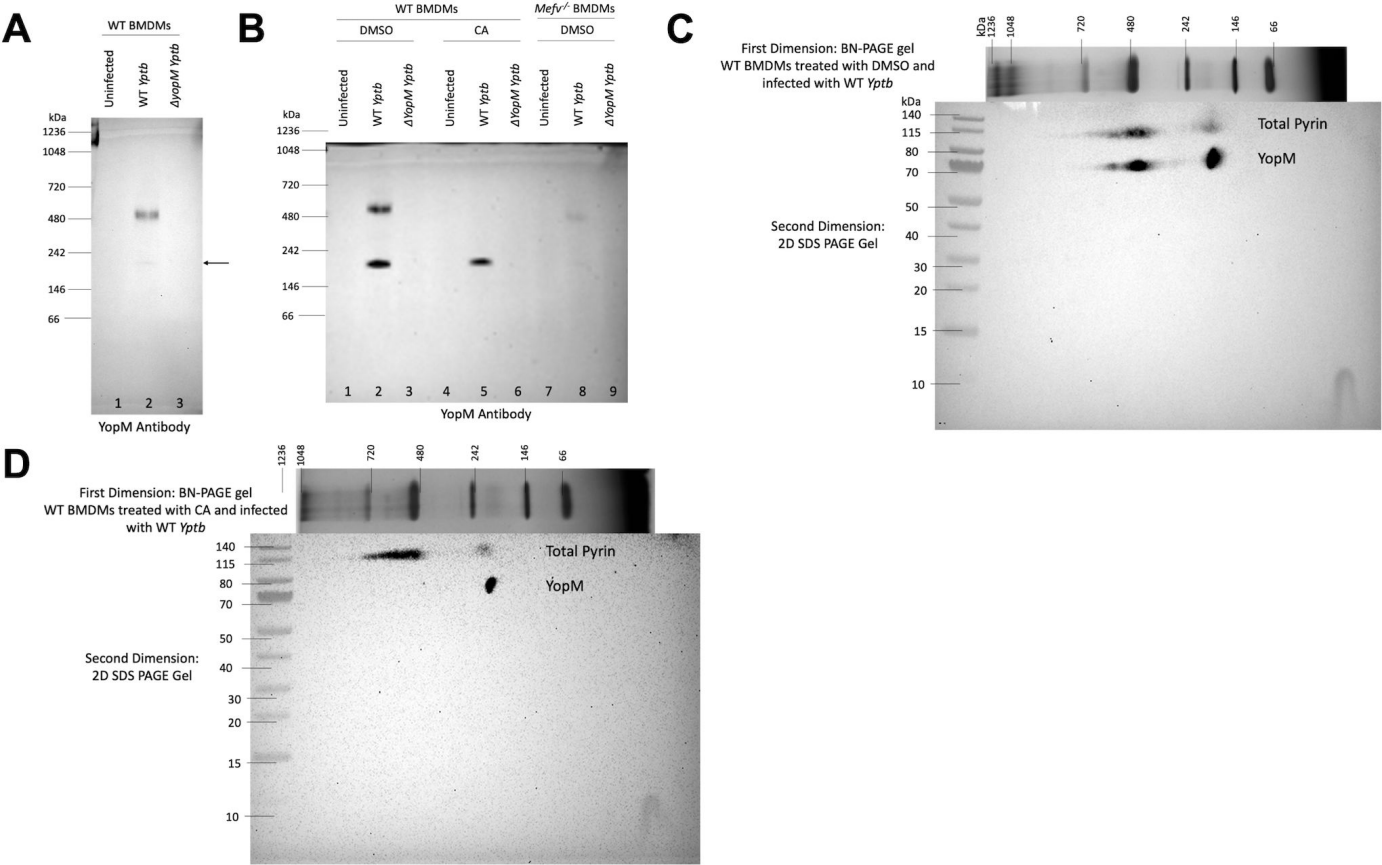
Detection of YopM interaction with pyrin oligomers by one-dimensional and two-dimensional BN-PAGE and immunoblotting. Untreated WT BMDMs (**A**) or WT or *Mefv^−/^* ^−^ BMDMs pretreated for 15 min and maintained in DMSO or 10 nM CA (**B–D**) were either left uninfected or infected with WT or *∆yopM Yptb* at an MOI of 30 for 90 min. Cell lysates were solubilized in 1% digitonin, separated by one-dimensional BN-PAGE, and immunoblotted for YopM (**A,B**). BN-PAGE MW standards are shown on the left, and tentative positions of YopM oligomers are shown on the right. Arrow in (**A**) indicates the band corresponding to YopM at pyrin’s dimer position. 1% digitonin samples from WT BMDMs treated with DMSO (**C**) or 10 nM CA (**D**) and infected with WT *Yptb* were separated in the first dimension by BN-PAGE after which the gel slice was isolated and analyzed by SDS-PAGE in the second dimension followed by immunoblotting for pyrin and YopM. MW markers for each dimension are indicated.

YopM can interact with pyrin as determined by co-immunoprecipitation (17, 18, 35). We wondered if YopM interacts with the different oligomeric forms of pyrin and if this interaction is sensitive to PPP inhibition. To determine if YopM is associated with pyrin oligomers, samples from untreated WT BMDMs left uninfected or infected with WT or *∆yopM Yptb* were separated by BN-PAGE and immunoblotted with antibody to YopM. We detected discrete bands of YopM at the dimer and higher-order oligomer pyrin position in the WT but not in the *∆yopM Yptb* samples (Fig. 5A, lanes 2 and 3). We then repeated the experiments using WT BMDMs untreated or treated with CA or untreated *Mefv^−/−^* BMDMs. In WT BMDMs, there was a YopM signal corresponding to dimeric pyrin in the DMSO and CA-treated WT *Yptb*-infected samples (Fig. 5B, lanes 2 and 5). The higher order oligomeric YopM signal around ~480 kDa seen with DMSO treatment was not present in the CA-treated sample (Fig. 5B, lane 5). In addition, there was a faint YopM signal around ~480 kDa in the *Mefv^−/−^* BMDMs infected with WT *Yptb* (Fig. 5B, lane 8). The YopM signal in lane 8 appeared to be of slightly lower molecular weight than the corresponding band in lane 2. Two-dimensional (2D) BN-PAGE immunoblot analysis of the DMSO-treated and WT *Yptb*-infected BMDM sample showed a good correspondence in the molecular weights of the YopM and oligomeric pyrin signals (Fig. 5C). These results strongly indicate that YopM interacts with dimeric and higher order oligomers of pyrin. Furthermore, when the CA-treated and WT *Yptb*-infected sample was subjected to 2D BN-PAGE analysis, the YopM signal around ~480 kDa disappeared but the band around ~242 kDa was still present (Fig. 5D), recapitulating the BN-PAGE results shown in Fig. 5B. Possible explanations for why YopM interaction with the higher order pyrin oligomer is sensitive to PPP inhibition and the presence of a weak YopM signal at this position in *Mefv* ^−/−^ BMDMs are discussed in Discussion section.

### Longer pretreatment with 100 nM OA reduces pyrin dephosphorylation and inflammasome assembly

**Fig 6:**
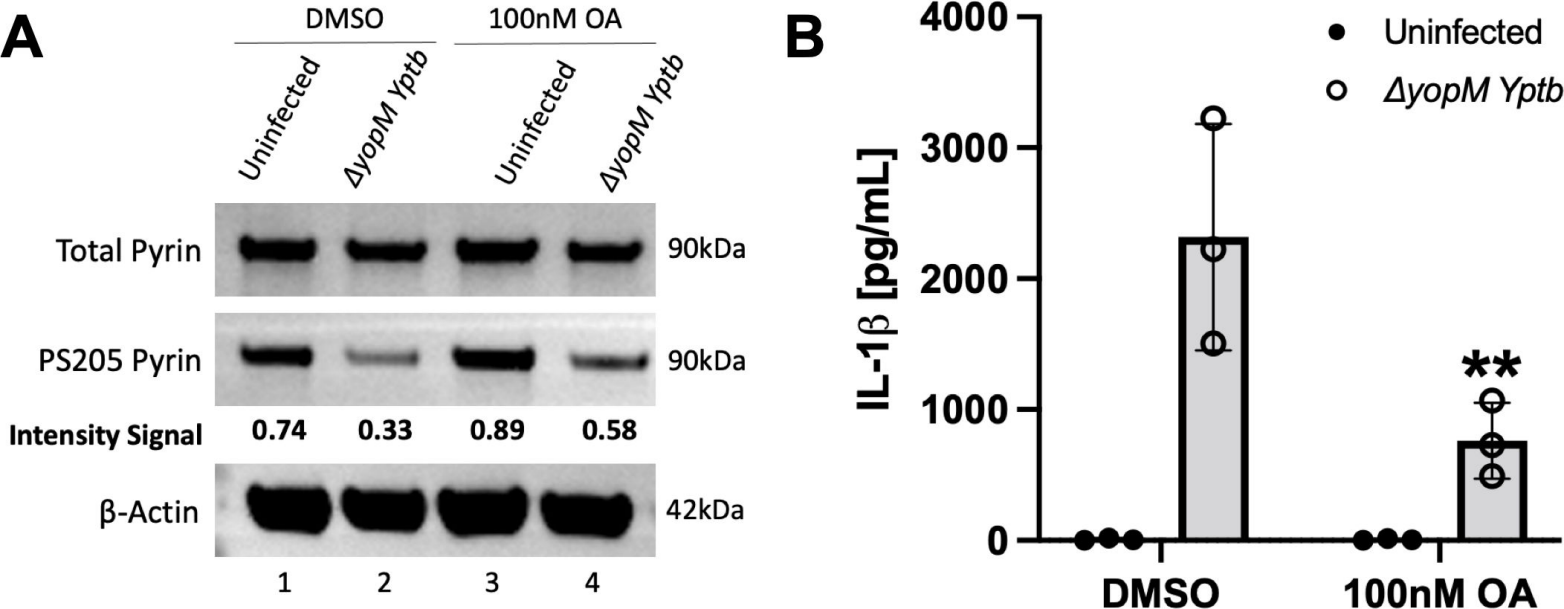
Longer pretreatment with 100 nM OA reduces pyrin dephosphorylation and IL-1β release in BMDMs infected with *∆yopM Yptb*. LPS-primed BMDMs were pretreated for 3 h and maintained with either DMSO or 100 nM OA and infected with the *∆yopM Yptb* at an MOI of 30 for 90 min. Treated uninfected BMDMs were analyzed in parallel. (**A**) Immunoblot analysis of BMDM lysates. Quantified immunoblot band intensity signals from one experiment representing PS205 pyrin/total pyrin are indicated below the respective blot image. (**B**) Mature IL-1β in supernatants as quantified by ELISA. Two-way ANOVA with Bonferroni post-hoc correction was applied to calculate the significance, and *P*-value of OA treatment as compared to its corresponding DMSO treated and *∆yopM* infected sample is indicated. *P* < 0.05 was considered significant; *P* < 0.01 (**). Each data group is presented as an average (error bars are standard deviation) of at least three independent experiments.

OA penetration inside cells is slow, and using a lower concentration of OA generally requires longer pretreatment time to achieve sufficient inhibitory levels inside the cell. In contrast, CA readily partitions inside cells (51 – 53). As shown in Fig. 1A and B, using a shorter pretreatment time for 100 nM OA was not effective at inhibiting pyrin’s dephosphorylation. We found that WT BMDMs pretreated for 3 h and maintained with 100 nM OA during infection with *∆yopM Yptb* showed reduced pyrin dephosphorylation (Fig. 6A) and a significant decrease in IL-1β release as compared to the DMSO control (Fig. 6B), whereas 15 min pretreatment of BMDMs with 100 nM OA did not have such an inhibitory effect on pyrin activation (Fig. 1A and B). Given the selectivity of 100 nM OA to preferentially inhibit PP2A/PP4 over PP1/PP5 (53), these results suggest that PP2A or PP4 could be involved in positively regulating pyrin. Since most serine/threonine phosphatase activities inside the cell are carried out by PP1 and PP2A (40, 41, 54), our results with TTN and longer pretreatment with OA in BMDMs allude to a role for PP2A in positive regulation of pyrin activation.

### siRNA knockdown implicates PP2A in the dephosphorylation of pyrin S205

**Fig 7:**
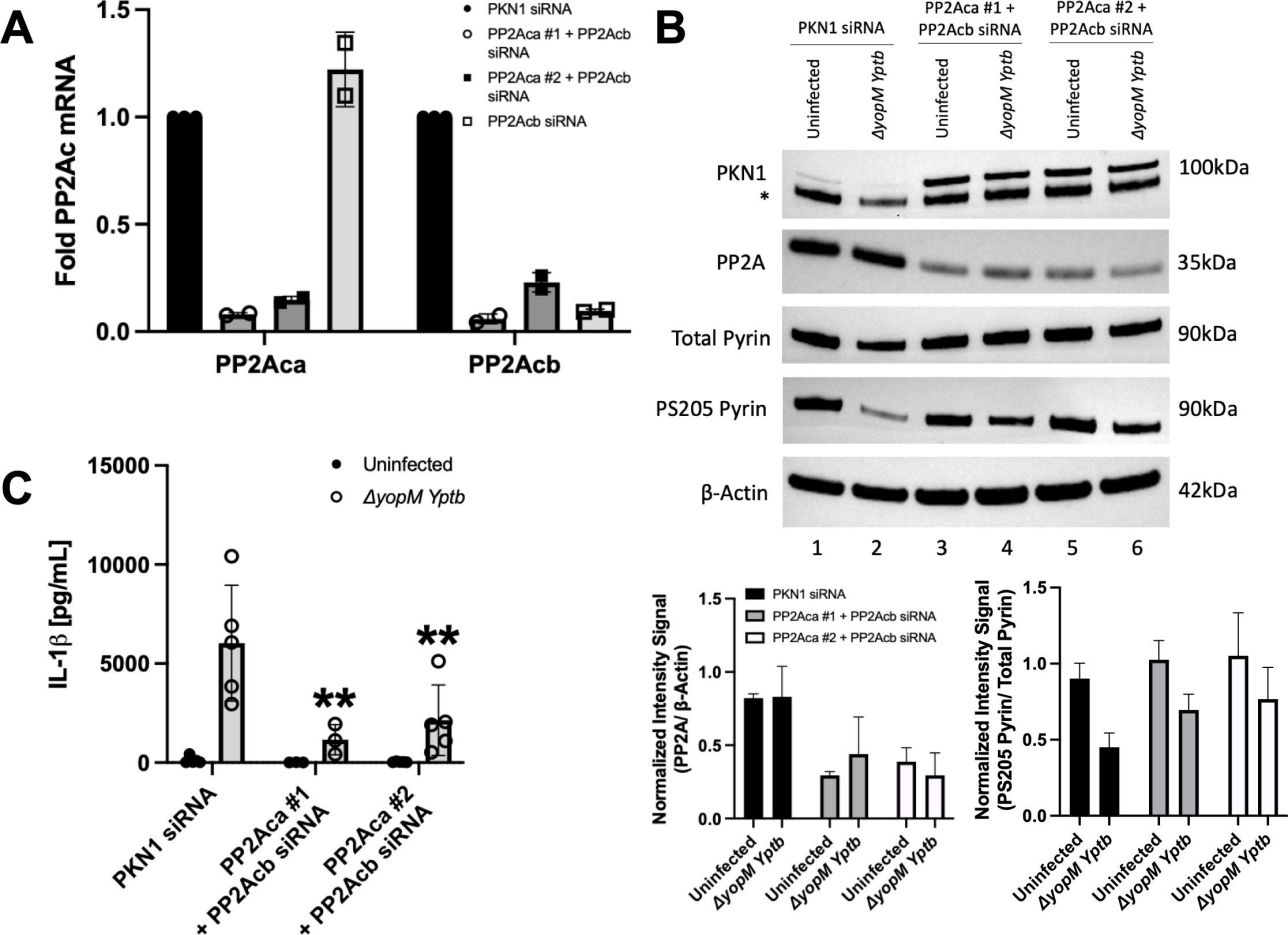
siRNA knockdown of PP2A in BMDMs inhibits pyrin S205 dephosphorylation and IL-1β release. WT BMDMs were electroporated with siRNAs targeting PKN1 (400 pmol) or PP2Aca + PP2 Acb (500 pmol each); 24 h after electroporation, the BMDMs were LPS primed; 48 h following electroporation, cells were either left uninfected or infected with *∆yopM Yptb* at an MOI of 30 for 90 min. (**A**) RT-qPCR analysis of mRNA transcripts of PP2Aca and PP2Acb in uninfected BMDMs. Results were normalized to Hprt mRNA levels. Samples with error bars represent standard deviation for an average of two independent experiments. (**B**) Immunoblot analysis of BMDM lysates. Asterisk (*) indicates bands for total pyrin on an immunoblot that was re-probed for PKN1. Quantified immunoblot band intensity signals are plotted and shown below the blot images to represent PP2A/β-actin and PS205 pyrin/total pyrin. Signals of bands from immunoblots from three independent experiments were averaged to calculate the mean and standard deviation. (**C**) Mature IL-1β in supernatants as quantified by ELISA. Two-way ANOVA with Bonferroni post-hoc correction was applied to calculate the significance, and *P*-value of samples as compared to the PKN1 siRNA and *∆yopM*-infected sample is indicated. *P* < 0.05 was considered significant; *P* < 0.01 (**). Each data group is presented as an average (error bars are standard deviation) of at least three independent experiments.

We next tested the effect of knocking down PP2A in BMDMs on pyrin inflammasome assembly. PP2A has two catalytic subunit isoforms, alpha (PP2Aca) and beta (PP2Acb), which only differ in a few amino acids from each other. We first tested two different siRNAs targeting the mRNA of the alpha catalytic subunit of PP2A (44). The siRNAs (1,000 pmol) were electroporated into WT BMDMs that were also LPS primed. PKN1 siRNA was used as a positive control for knockdown (17, 34) and a negative control for the PP2A siRNAs. RT-qPCR showed that there was significant and selective knockdown of PP2Aca mRNA with both siRNAs as compared to the positive control and PP2Acb analyzed in parallel (Fig. S5A). The siRNA electroporations were then repeated, and LPS-primed BMDMs were left uninfected or infected with *∆yopM Yptb*. Immunoblotting showed that there was full depletion of PKN1 with the corresponding siRNA but only an apparent ~50% knockdown of PP2A with the siRNAs #1 and #2 (Fig. S5B). Since the PP2A antibody used detects both isoforms, the remaining signal detected could have corresponded to PP2Acb. Upon *∆yopM* infection, there was no inhibition of dephosphorylation of pyrin at S205 with either PP2Aca siRNAs (Fig. S5B). However, PP2Aca #2 siRNA decreased IL-1β secretion (unpublished observation), which could indicate that this siRNA has an off-target effect when used at a high concentration. A PP2Acb siRNA when tested by itself at 500 pmol selectively knocked down the corresponding mRNA transcripts and did not decrease IL-1β release as compared to the control (Fig. 7A; Fig. S4C, respectively). We next tested the effect of a combined PP2A alpha and beta catalytic subunit knockdown. BMDMs were electroporated with PKN1 or PP2Aca and PP2Acb siRNAs (500 pmol each) and LPS primed as before. RT-qPCR showed that there was a decrease in the mRNA transcripts of both PP2A subunits (Fig. 7A). Immunoblotting showed a greater depletion of PP2A protein as compared to knocking down the alpha subunit alone (compare Fig. S5B and Fig. 7B). Upon *∆yopM* infection, PS205 pyrin was maintained, and there was a significant decrease in IL-1β secretion with both siRNA combinations tested (Fig. 7B and C, respectively). Together, these results implicate PP2A alpha and beta subunits as acting redundantly to dephosphorylate pyrin S205.

## DISCUSSION

In this study, we aimed to understand if PPP activity is important for the positive regulation of inflammasome assembly by oligomeric pyrin. There are seven different PPPs in the mammalian cell: PP1, PP2A, PP2B (PP3), PP4, PP5, PP6, and PP7. In the human genome, there are ~428 Ser/Thr kinases but only ~30 Ser/Thr phosphatases (54). The remarkable diversity of PPP substrates, substrate recognition, and cellular localization is achieved by the association of catalytic subunits with noncatalytic subunits to form multimeric holoenzymes (40, 41). The active sites of these phosphatases are highly similar, and most phosphatase inhibitors that bind to the PPP active sites are broadly specific for this reason.

CA is a relatively nonspecific and hydrophobic PPP inhibitor. It can easily partition through cell membranes and potently inhibits most PPPs except for PP2B and PP7 (53). CA was found to inhibit both murine and human pyrin inflammasome activation. Using 10 nM CA, we obtained evidence that PPP activity is important for pyrin S205 dephosphorylation and inflammasome assembly in BMDMs in response to RhoA inactivation by TcdB or YopE/T during *∆yopM Yptb* infection. The same CA treatment also prevented TecA-dependent dephosphorylation of pyrin S242 in THP-1 cells infected with *B. cenocepacia*. It has been shown previously in mouse peritoneal macrophages that 50 nM CA prevented the activation of NLRP3, AIM2, and NLRC4 inflammasomes, indicating that PPP activity can be important for inflammasome activation in general and is perhaps acting at a latter step (45). In our experiments with primary monocytes, 40 nM CA also prevented NLRP3 inflammasome activation. We observed that 10 nM CA prevented the infection-induced pro-IL-1β synthesis in THP-1 cells infected with *B. cenocepacia* illustrating that results with this inhibitor need to be carefully controlled and interpreted since it can block multiple steps in an inflammasome pathway. Moreover, 40 nM CA was also found to reverse the effect of a PKN inhibitor and potently inhibit pyrin activation in FMF patient monocytes. These cells have previously been demonstrated to exhibit a lower activation threshold for inflammasome activation as compared to monocytes from healthy donors, and pyrin dephosphorylation has been shown to be sufficient to trigger inflammasome assembly in FMF monocytes (38, 39). It is possible that in the FMF disease context, a balance between the PKN kinases and a PPP regulates the inflammasome assembly and inhibition of either shifts of the equilibrium in favor of the other protein. Results from our experiments with treatment of BMDMs with CA and *∆yopK Yptb* mutant infection showed that there was a nonsignificant decrease in IL-1β release which can be attributed to the inhibition of NLRP3 but not of the NLRC4 inflammasome. Luheshi et al. used a higher CA concentration of 50 nM (45), which could explain why we didn’t observe the inhibition of NLRC4 inflammasome in response to *∆yopK* infection with 10 nM CA treatment.

We were also able to show using BN-PAGE that murine pyrin forms oligomers consisting of dimers and higher order oligomers that are phosphorylated on S205 when pyrin is inactive. Due to the insolubility of active pyrin in nonionic detergent, we were unable to determine if dephosphorylation changes the oligomeric status of pyrin. However, PPP inhibition with CA resulted in the appearance of a very high molecular weight mystery oligomer which indicates that phosphorylation increases the oligomerization of pyrin. Pyrin belongs to the TRIM family of proteins, and Trim5α, which is involved in HIV viral capsid recognition, is known to form dimers and higher order lattices consisting of dimers of dimers or trimers of dimers (28). The higher order murine pyrin oligomers (~480–720 kDa) that we observe on BN-PAGE immunoblots could represent dimers of dimers or trimers of dimers similar to those formed by Trim5α. It has been previously shown through crosslinking studies in THP-1 cell lysates that human pyrin can form trimers that is mediated through its coiled-coil domains (30). In the same study, it was also shown that the B-box domain of human pyrin interacts with the N-terminal pyrin domain to sequester it and prevent the interaction with ASC (30). However, Weinert et al. reported that a truncated version of human pyrin forms an antiparallel dimer *in vitro* through the interaction of coiled-coil domains (29). These discrepancies in trimer vs dimer formation by human pyrin could be attributed to experimental and technical differences. It is expected that the regulation of human and murine pyrin by oligomerization would be conserved but due to structural differences, it is possible that murine pyrin forms dimers and human pyrin forms trimers through their respective coiled-coil domains. To rule out technical differences, it would be informative to investigate the oligomerization of human pyrin by BN-PAGE instead of crosslinking. The phosphorylation status of human pyrin oligomers should also be determined.

CA treatment resulted in an upshift in molecular weights of PS205 and total murine pyrin bands in all conditions on BN-PAGE immunoblots, as well as on some SDS-PAGE immunoblots. In addition, CA treatment of THP-1 cells resulted in the hyperphosphorylation of S242 in human pyrin. This indicates that a PPP acts constitutively on the second linker site in pyrin to dephosphorylate it, and when this PPP is inhibited, the second serine becomes hyperphosphorylated. These results suggest that one of the linker sites can be hypophosphorylated yet pyrin remains inactive. This finding is surprising given the data that phosphorylation of the second linker site is critical for the negative regulation of pyrin, based on alanine substitution of this residue in ectopically expressed pyrin variants (34, 35). In addition, the S242R substitution causes PAAND, which is a clinical presentation distinct from FMF, in which the pyrin variant is considered constitutively active (36). Pull-down experiments in the PAAND study also showed that 14-3-3 proteins are not bound to the S242R variant and that pyrin is dephosphorylated (36). Jeru et al. showed previously that an S208A pyrin variant has reduced 14-3-3 binding, while the S242A mutant abolishes 14-3-3 binding (55). This suggested that 14-3-3 binding could follow a gatekeeper hypothesis in which PS242 is the dominant site that promotes 14-3-3 binding, and PS208 is a secondary site (55). These findings may indicate that the hypophosphorylation of the second linker site is sufficient to keep pyrin oligomers inactive. This could facilitate fast dephosphorylation of the hypophosphorylated second sites, resulting in rapid activation of pyrin in response to RhoA inactivation by pathogens. A caveat of this concept is our limitation of not being able to measure the phosphorylation status of S241 in murine pyrin under the conditions used.

We were also able to use 1D and 2D BN-PAGE and immunoblotting to detect the interaction of murine pyrin with YopM during WT *Yptb* infection of BMDMs and found that this interaction was sensitive to PPP inhibition. The fact that YopM targets the oligomers argues that oligomeric pyrin is important for pyrin inflammasome activation. We hypothesize that the faint YopM signal on BN-PAGE immunoblots from the *Mefv^−/−^* BMDMs infected with WT *Yptb* could result from the self-oligomerization of the YopM protein. The YopM protein from *Y. pestis* is known to form tetramers *in vitro* (37, 56), so it is conceivable that the *Yptb* YopM protein can form higher order oligomers on its own in *Mefv^−/−^* BMDMs. One explanation for the selective loss of the YopM signal at the position of the higher order oligomers from the CA-treated and WT *Yptb-*infected samples is that the affinity of YopM binding to pyrin is reduced by hyperphosphorylation.

OA in comparison to CA has a greater inhibitory constant to bind to the catalytic subunit of PP2A than of PP1 and is reported to enter cells slowly (53, 57). Our results indicated that 15 min pretreatment with 1,000 nM OA is sufficient to inhibit S205 pyrin dephosphorylation and IL-1β release in BMDMs upon RhoA inactivation. Stutz et al. reported that PP2A is important for NLRP3 activation through dephosphorylation using the same conditions and concentration of OA (44). We only observed a reduction in S205 pyrin dephosphorylation and a significant decrease in IL-1β secretion in response to *∆yopM* infection when BMDMs were pretreated with 100 nM OA for 3 h but not for 15 min, which can be explained by the slow entry of the inhibitor. We hypothesize from these results that PP2A plays a role in the dephosphorylation of S205 and positive regulation of the pyrin inflammasome in BMDMs.

PP2A, along with PP1, carries out most serine/threonine dephosphorylation events in cells and partakes in many essential activities for the cell (40, 41). PP2A is abundantly present in cells and forms heterotrimers consisting of a catalytic subunit, a scaffolding A subunit, and a regulatory B subunit (40, 41). There are two isoforms of the catalytic and scaffolding subunits, and four isoforms of the regulatory subunits, which account for the diverse activities and substrates regulated by PP2A (54). Since it is an essential protein for the cell and very likely has a long half-life that explains why we were unable to fully deplete it in the cell. The alpha and beta catalytic subunits of PP2A only differ by a few amino acids, and PP2Aca is more abundantly present in cells than PP2Acb. Our results indicated that siRNAs targeting PP2Aca did not reduce S205 pyrin dephosphorylation in BMDMs infected with *∆yopM*. However, a combined knockdown of both catalytic subunits of PP2A showed that S205 pyrin phosphorylation was maintained, and there was a significant decrease in IL-1β secretion upon *∆yopM* infection. These results indicate that the two catalytic subunits of PP2A act redundantly to dephosphorylate the pyrin linker. This is distinct from the case of NLRP3 where the knockdown of the alpha catalytic subunit of PP2A alone was sufficient to prevent the assembly of the inflammasome (44). To confirm our results, additional siRNAs targeting the alpha and beta catalytic subunits of PP2A should be tested. In addition, the impact of PP2A knockdowns on PS241 in murine pyrin and PS208/242 in human pyrin needs to be investigated. The phosphorylation status of murine PS241 and human PS208 in pyrin could be investigated using immunoprecipitation combined with mass spectrometry or the use of pan-specific antibodies to phosphoserine. Additionally, the use of pyrin variants with amino acid changes that ablate or mimic serine phosphorylation (31) could be helpful to corroborate the siRNA knockdown data. Moreover, in a broader disease context, the effect of PP2A knockdown on FMF pyrin activation should also be explored because it may offer insights for new therapeutic strategies.

PPPs recognize and bind to regulatory proteins or to substrates via short linear motifs (SLiMs) (40, 41). For example, the B56 regulatory subunit of PP2A can bind through LxxIxE motifs to its substrates (40, 41). Interestingly, murine pyrin contains two predicted LxxIxE motifs in the coiled-coil domain, and human pyrin has one predicted LxxIxE motif in the linker. Since both murine and human pyrin have a predicted PP2A-B56 regulatory motif, it is possible that PP2A regulates both forms through a conserved mechanism. We are currently testing the importance of the predicted SLiM motifs in murine and human pyrin. However, it should be noted that the majority of PP2A substrates do not have functional SLiMs but instead interact with SLiM-containing adaptor proteins that connect them to PP2A-B56 (41). We have not ruled out the possible roles of other PPPs in pyrin dephosphorylation and inflammasome assembly, and additional approaches will likely be needed to address this possibility. Finally, it remains to be determined if the activity of PP2A is upregulated upon RhoA inactivation, or if these enzymes simply constitutively counterbalance PKN. In the case of PP2A activating NLRP3 by dephosphorylation (44), there is evidence that Bruton tyrosine kinase negatively regulates PP2A by phosphorylation of tyrosine 307 (58). It is theoretically possible that PKN could negatively regulate PPP activities by phosphorylation, and additional experiments are needed to address this possibility.

## MATERIALS AND METHODS

### Bacterial strains

*Y. pseudotuberculosis* (*Yptb*) 32777 (serogroup O1) strains used in this study are as follows: wild-type (59), *∆yopM* (60), *∆yopK* (61), *yopE^R144A^ΔyopM,* and *yopT^C139A^ΔyopM* (17). *Yptb* was grown at 28°C on Luria broth (LB) agar plates or with shaking in LB broth, supplemented with antibiotics when necessary. The *B. cenocepacia* strains used were AU1054 (WT) and *∆tecA* mutant (62). *B. cenocepacia* was grown at 37°C on LB agar plates or with shaking in LB broth.

### Bone marrow isolation and cell culture conditions

BMDMs were obtained from the femurs of 8-week-old WT C57BL/6 mice (Jackson Laboratories) and/or *Mefv^−/^* ^−^ mice (63) as previously described (19). Cells were grown at 37°C in a humidified incubator in macrophage growth media (MGM) made up of Dulbecco’s modified Eagle Medium (DMEM) plus GlutaMAX (Gibco) containing 10% heat-inactivated fetal bovine serum (FBS) (GE HealthCare), 10% L929 cell-conditioned medium, 1 mM sodium pyruvate (Gibco), and 10 mM HEPES (Gibco) for a week. On day 7, differentiated macrophages were divided into six-well plates at a density of 0.8 × 10^6^ cells/well in a total volume of 3 mL in MGM diluted to contain 10% L929 cell-conditioned medium (MGM 10/10) and primed for ~18 h with 100 ng/mL O26:B6 *Escherichia coli* LPS (Sigma).

### BMDM infection, intoxication, and inhibitor treatments

For BMDM infections, *Yptb* strains were grown overnight in LB at 28°C. The following day, cultures were diluted 1:40 in LB containing 20 mM sodium oxalate and 20 mM MgCl2 and grown at 28°C for 1 h, then shifted to 37°C for 2 h. Cultures were centrifuged and the bacterial pellets were resuspended in phosphate-buffered saline (PBS). Bacterial suspensions were then diluted in media lacking FBS and containing 10% L929 cell-conditioned medium (MGM 0/10). LPS-primed BMDMs were washed once with PBS and left uninfected or infected in MGM 0/10 with *Yptb* strains cultured under conditions as described above at an MOI of 30. Plates were centrifuged for 5 min at 106 *×g* to facilitate contact between *Yptb* and macrophages. Plates were then incubated at 37°C in 5% CO_2_. In experiments where the glucosyltransferase toxin TcdB from *Clostridium difficile* (List Biological Laboratories, Inc.) was used as a positive control for pyrin inflammasome activation, BMDMs were intoxicated with TcdB at a concentration of 0.1  µg/mL for 90 min in MGM 0/10 media.

In experiments where phosphatase inhibitors were used and BMDMs were pretreated for 15 min with either 1.4 mM DMSO as vehicle control or calyculin A (10 nM diluted from 100 µM stock, LC Laboratories), cyclosporin A (1 µM diluted from 1 mM stock, Sigma), okadaic acid (100 nM or 1 µM diluted from 1 mM stock, Tocris), or tautomycetin (1 µM or 2 µM diluted from 1 mM stock, Tocris) in MGM 0/10, MGM 10/10 was used for BMDMs pretreated for 3 h with 100 nM okadaic acid. For infection following pretreatment, MGM 0/10 media containing bacteria and DMSO or the respective phosphatase inhibitors was added to the BMDMs, followed by centrifugation and incubation as above. Post-infection, cell supernatants were collected and processed for IL-1β detection by ELISA, and cell lysates were prepared and processed for immunoblot analysis (see below).

### THP-1 cells, infection, and inhibitor treatment

THP-1 cells (ATCC TIB-202) were grown in filter-sterilized RPMI 1640 media (Cat. No 30–2001) containing 10% heat-inactivated fetal bovine serum and 0.05 mM 2-mercaptoethanol in 25 cm^2^ flasks. When confluent, the THP-1 cells were expanded in 75 cm^2^ flasks. No cells were used that were passaged more than 30 times. For infections, THP-1 cells were seeded at a density of 0.8 × 10^6^ cells per well in 1.5 mL of media in 6-well plates and pretreated with 1.4 mM DMSO vehicle control or 10 nM calyculin A for 15 min. Overnight (16 h) cultures of *B. cenocepacia* were subcultured 1:100 in fresh LB on infection day and shaken at 37°C until the cultures reached mid-log phase (OD_600_ = 0.300). Cultures were then centrifuged, the LB was removed, and the bacterial pellets were resuspended in PBS and then diluted to an MOI of 20 in warmed RPMI 1640 media without FBS and containing DMSO or calyculin A; 1.5 mL of supplemented media was added to each well and mixed by pipetting gently up and down. Following incubation for 3 h at 37°C, the contents of each well were collected and centrifuged for 10 min at 14,000 rpm and 4°C. The cell pellet was collected and lysed for immunoblot analysis as described below.

### Primary monocytes and inhibitor treatment

Primary monocytes purified as described (39) from four healthy donors and three FMF patients carrying the p.M694V/p.M694V mutation were seeded into 96-well plates at 5 × 10^3^ cells/well, in RPMI 1640, GlutaMAX medium (Thermo Fisher Scientific) supplemented with 10% fetal calf serum (Lonza). Cells were incubated for 2.5 h in the presence of LPS (10 ng/mL, InvivoGen) and, when indicated, pretreated for 30 min with calyculin A (40 nM, Sigma 208851), followed by treatment with UCN-01 (12.5 µM, Sigma) or nigericin (5 µg/mL, InvivoGen) for 90 min. Following the incubation, cells were centrifuged, and supernatants were collected for IL-1β ELISA.

### U937 cell line, genetic manipulation, and inhibitor treatment

The protocol for genetically manipulating the human myeloid cell line U937 (CelluloNet, Lyon, France) was followed as described previously in reference (39). Cells were grown in RPMI 1640 medium with glutaMAX-I supplemented with 10% (vol/vol) FCS, 2 mM L-glutamine, 100 IU/mL penicillin, and 100 µg/mL streptomycin (Thermo Fisher Scientific). *MEFV* ^KO^ cell lines generated by CRISPR/Cas9-mediated gene invalidations have been previously described (39, 64). p.M694V *MEFV* was cloned into the GFP-expressing plasmid pINDUCER21 (65) under the control of a doxycycline-inducible promoter through the pENTR1A (Invitrogen) vector using a synthetic DNA fragment (GeneArt) encoding the p.M694V Pyrin protein. Lentiviral particles were produced in 293T cells using pMD2.G and psPAX2 (from Didier Trono, Addgene plasmids #12259 and #12260), and pINDUCER-21 p.M694V Pyrin. U937 *MEFV* ^KO^ cell lines were transduced by spinoculation and sorted at day 7 post-transduction based on GFP expression on an Aria cell sorter. Pyrin expression was induced by treatment with doxycycline (1 µg/mL) for 16 h before stimulation. To assess IL-1β release, 5 × 10^4^ U937 cells per well were treated with 100 ng/mL of phorbol 12-myristate 13-acetate (PMA; InvivoGen) for 48 h. The medium was changed, and cells were primed with LPS (50 ng/mL, InvivoGen) for 2.5 h and treated with UCN-01 (12.5 µM, Sigma) with or without a 30 min pretreatment with calyculin A (40 nM, Sigma). Supernatant was collected at 3 h post-UCN-01 treatment.

### Real-time cell death assay

U937 *MEFV* ^KO^ cells expressing the p.M694V *MEFV* variant were seeded at 5 × 10^4^ per well before stimulation in a black 96-well plate (Costar, Corning) with propidium iodide (PI, Sigma) at 5 µg/mL. Three technical replicates per condition were done. Cells were pretreated with calyculin A (Sigma, 208851) at 40 nM for 30 min and then treated with UCN-01 (Sigma, U6508) at 12.5 µM. Real-time PI incorporation was measured every 5 min for 6 h post-UCN-01 addition on a fluorimeter (Tecan) using the following wavelengths: excitation 535 nm (bandwidth 15 nm) and emission 635 nm (bandwidth 15 nm) (66, 67). Cell death was normalized [previously described in reference (39)], using PI incorporation in monocytes treated with 1% Triton X-100 for 15 min (=100% cell death). As a further correction, the first time point of the kinetics was set to 0.

### Protein analysis by SDS-PAGE and immunoblotting

To obtain cell lysates, BMDMs and THP-1 cells were lysed in mammalian protein extraction reagent (MPER, Thermo Scientific) along with cOmplete Mini (Roche) protease inhibitor and PhosSTOP (Roche) phosphatase inhibitor. The lysis buffer for THP-1 cells was supplemented with 10 nM calyculin A. Protein concentration was normalized after measurement with a bichinonic acid assay (BCA, Thermo Fisher Scientific), and 3–10 μg of total protein cell lysates was run on 4–12% NuPAGE Bis-Tris SDS-PAGE gels (Invitrogen by ThermoFisher Scientific) and transferred to polyvinylidene difluoride membranes (ThermoFisher Scientific) using an iBlot 2 gel transfer device (Life Technologies). Membranes were blocked in 5% nonfat dairy milk (add company) and incubated with primary antibodies overnight. Primary antibodies used in this study were rabbit-anti-mouse monoclonal total pyrin antibody (1:1,000 dilution, ab195975; abcam), rabbit-anti-mouse pyrin polyclonal [1:1,000 dilution (63)], rabbit-anti-mouse phospho-serine 205 monoclonal antibody (1:1,000 dilution, ab201784; abcam), rabbit-anti-mouse phospho-serine 241 monoclonal antibody (1:1,000 dilution, ab201784; abcam), rabbit-anti-mouse/human IL-1β (1:1,000 dilution, number 12242; Cell Signaling), rabbit-anti-mouse/human polyclonal β-actin (1:1,000 dilution, number 4967; Cell Signaling), mouse-anti-mouse/human/rat monoclonal PP1a (1:1000 dilution, number MA5-17239; ThermoFisher Scientific), rabbit-anti-*Y. pestis* polyclonal YopM (1:1,000 dilution; provided by Susan Straley), mouse-anti-mouse monoclonal PKN (1:1,000 dilution, number 393344; Santa Cruz Biotechnology), rabbit-anti-mouse monoclonal PP2A (1:1,000 dilution, #2038; Cell Signaling), and rabbit-anti-mouse GSDMD (1:1,000 dilution, ab209845; Abcam). Horseradish peroxidase-conjugated anti-rabbit or anti-mouse antibody (Jackson Laboratory) were used as secondary antibodies. Protein bands bound by antibodies were visualized using chemiluminescent detection reagent (GE Healthcare) on an iBright FL1500 (ThermoFisher Scientific). Immunoblot quantification was performed using the iBright analysis software, and “local corrected volume” in the software was used to represent signal intensity for a band where indicated.

### Protein analysis by BN-PAGE and immunoblotting

BN-PAGE immunoblotting was performed as described by Kofoed and Vance (50). All reagents for this procedure were obtained from Invitrogen except as stated. BMDMs were lysed in non-denaturing 1X Native-PAGE Novex sample buffer with the indicated concentrations of either digitonin (5% stock) or DDM (10% stock), MPER (Thermo Scientific), or RIPA lysis or extraction buffer (Thermo Scientific), along with cOmplete Mini (Roche) protease inhibitor and PhosSTOP (Roche) phosphatase inhibitor. Lysates were centrifuged at 20,817 *×g* for 10 min to obtain soluble and insoluble fractions. In some experiments, the insoluble fractions of 1% digitonin lysates were analyzed by SDS-PAGE and immunoblotting as described above. Protein concentrations of soluble fractions were normalized after measurement with a bichinonic acid assay (BCA, Thermo Fisher Scientific), and 10 µg or more of total protein cell lysates was mixed with NativePAGE G-250 additive and loaded on 4–16% NativePAGE Novex 3–12% Bis-Tris gels. NativeMark unstained protein standard was used for molecular weight standard visualization. XCell SureLock Mini-Cell was used for running the gels. Samples were run at room temperature (RT) at constant 150 V for 105–120 min using prechilled buffers. Dark blue cathode buffer (10 mL NativePAGE running buffer, 10 mL NativePAGE cathode additive, 180 mL deinonized water) was used for the inner chamber until samples had run up to one-third of the gel length after which the buffer was replaced with light blue buffer (10 mL NativePAGE running buffer, 1 mL NativePAGE cathode additive, 189 mL deinonized water) for the remainder of the run. The outer chamber was filled with NativePAGE running buffer. Gels were soaked in 2× NuPAGE transfer buffer (Invitrogen) for 15 min at shaking at RT. Proteins were transferred to polyvinylidene difluoride membranes (ThermoFisher Scientific) using the template program P3 (20 V constant, 7 min) and an iBlot 2 gel transfer device (Life Technologies). Post-transfer membranes were incubated in 8% acetic acid for 15 min with shaking, followed by ponceau S staining for 5 min. Membranes were washed with distilled water for 5 min to remove excess ponceau S stain, then air-dried, reactivated by immersion in methanol, followed by blocking in 5% nonfat dry milk. Primary and secondary antibodies used are mentioned above.

### Protein analysis by 2D BN-PAGE and immunoblotting

The procedures used followed a previously described protocol (50). Samples were prepared as described above and separated on NativePAGE Novex 3–12% Bis-Tris gels. Gel slices containing samples of interest were excised, trimmed to be 3 mm shorter in length than the size of the 2D well in NuPAGE 4–12% Bis-Tris 2D Gels, then submerged and shaken at RT in 1× NuPAGE sample buffer containing 0.1M DTT. Gel slices were then microwaved for ~20 s to denature proteins followed by cooling with shaking at 10 min at RT. Slices were then fitted into the 2D well and SDS-PAGE immunoblotting was performed as described above.

### siRNA knock-down

siRNAs targeting *Pkn1* (s115927), *PP2Aca* (#1: s72067, #2: s72066), and *PP2Acb* (s72069) were obtained from Invitrogen. For siRNA gene knockdown experiments, 400 pmol of *Pkn1* and 1,000 pmol of *PP2Aca* siRNAs, and for combined PP2Ac knockdown, 500 pmol each of PP2Aca siRNA and PP2Acb siRNA, were transfected into BMDMs (1 × 10^6^ cells) by electroporation using the Neon Transfection System (Invitrogen) and electrical parameters of 1,400 V, 20 ms, and two pulses. BMDMs were then replated in 6-well plates and grown in MGM 10/10 media; 24 h following transfection, media was changed to fresh MGM 10/10, and LPS was added for overnight incubation; 48 h following transfection, BMDMs were infected with *∆yopM Yptb* as described above.

### Quantitative real-time PCR (RT-qPCR)

At 48 h following transfection, uninfected BMDMs were lysed with TRIzol reagent (Invitrogen) to isolate RNA according to the manufacturer’s instructions; 500–1,000 ng of RNA was used to make cDNA using SuperScript IV First-Strand Synthesis System (Invitrogen). Approximately ~5 ng of cDNA was quantified using PowerUp SYBR Green Master Mix (Invitrogen) in a BioRad iCycler. Primer specificity was checked by melt-curve analysis. Expression of target genes was normalized to the expression of the housekeeping gene Hprt. For fold change analysis, data were transformed using the “relative standard curve method and comparative threshold cycle (Ct) method (ΔΔCt)” as described by Applied Biosystems. Published primer sequences were used for qPCR analysis (44), and the sequences are as follows: Hprt, forward 5′-TGAAGT ACTCATTATAGTCAAGGGCA-3′ and reverse 5′-CTG GTGAAAAGGACCTCTCG-3′; Ppp2ca, forward 5′-TCTTCCTCTCACTGCCTTGGT-3′ and reverse 5′-CAG CAAGTCACACATTGGACCC-3′; Ppp2cb, forward 5′-AAGGCGTTCACCAAGGAGCT-3′ and reverse 5′- ACAGCGGACCTCTTGCACAT-3′.

### IL-1β quantification

IL-1β in BMDM supernatants was quantified using a murine (MLB00C; R&D Systems) and human (R&D Systems) ELISA kit by following the manufacturer’s instructions.

### Statistical analysis

GraphPad Prism was used to perform statistical analyses. IL-1β ELISA data were analyzed by one-way or two-way analysis of variance (ANOVA; Bonferroni multiple-comparison test) or by Kruskal-Wallis test (Dunn’s multiple comparison test) as specified.

## SUPPLEMENTAL MATERIAL

Supplemental figures mbio.02066-23-s0001.pdf Fig. S1-S5.

ASM does not own the copyrights to Supplemental Material that may be linked to, or accessed through, an article. The authors have granted ASM a non-exclusive, world-wide license to publish the Supplemental Material files. Please contact the corresponding author directly for reuse.
