# Supplementary material for: Phosphoprotein phosphatase activity positively regulates oligomeric pyrin to trigger inflammasome assembly in phagocytes
Source: mBio. 2023 Oct 3;14(5):e02066-23. doi: 10.1128/mbio.02066-23 (PMC10653879; doi:10.1128/mbio.02066-23)
Supplement: Supplemental figures — Fig. S1-S5. [file mbio.02066-23-s0001.pdf]

Supplemental Figures

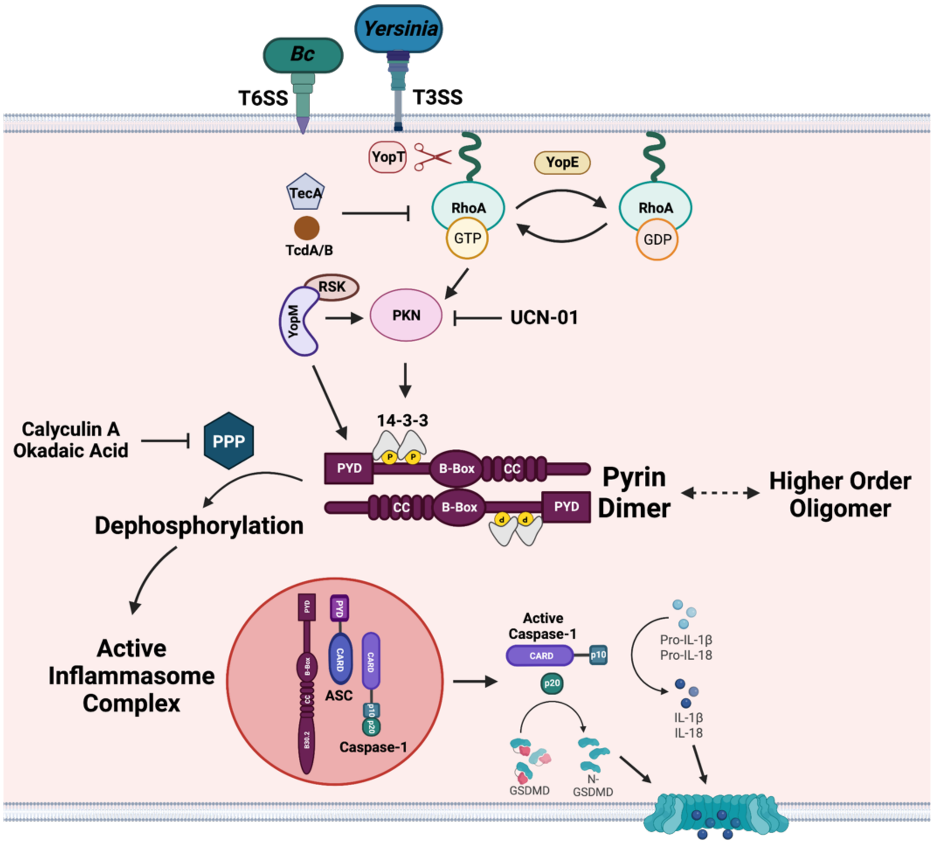

Fig. S1. Schematic of pyrin inflammasome pathway regulation by bacterial effectors and toxins, RhoA-PKN signaling, oligomerization, and PPP dephosphorylation, with PKN and PPP inhibitors indicated (see text for detail). Figure created with BioRender.com.

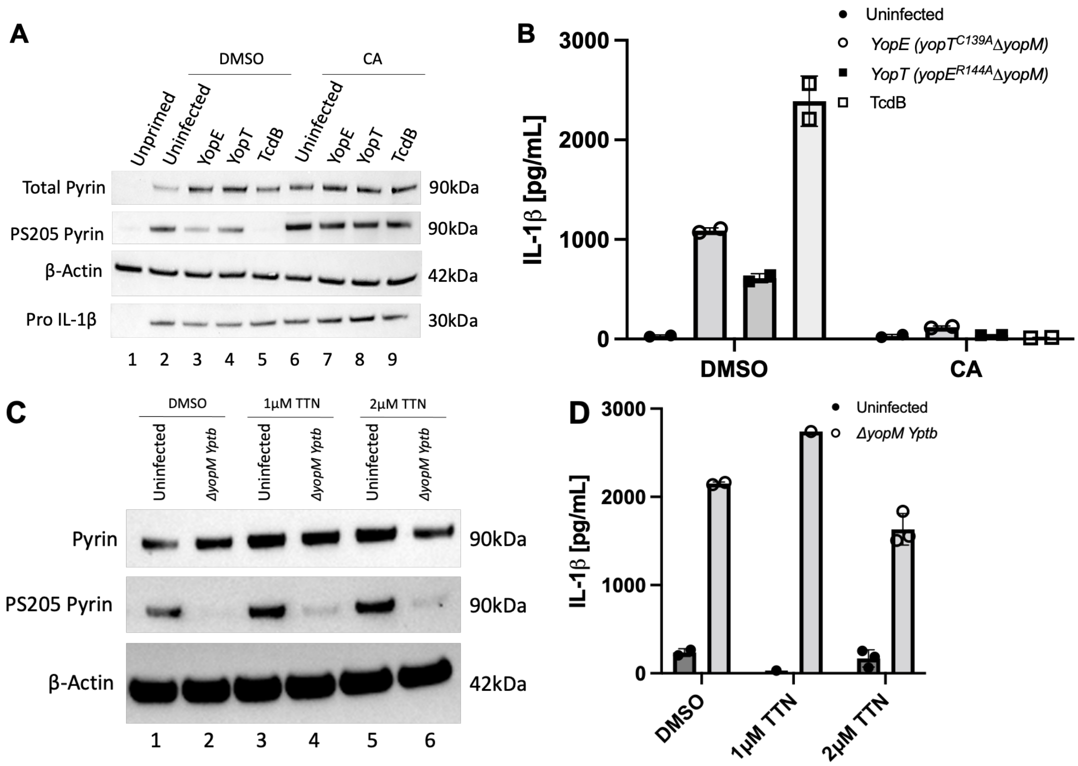

Fig.S2. PPP inhibition with CA but not TTN prevents pyrin S205 dephosphorylation and reduces IL-1β release in BMDMs infected with Δ*yopM Yptb* strains. LPS-primed BMDMs were pretreated for 15mins and maintained with either (A,B) 10nM calyculin A (CA), or (C,D) 1 or 2μM tautomycetin (TTN). In (A,B) cells were left unintoxicated or intoxicated with TcdB for 90 mins, or infected with either Δ*yopM Yptb* YopE (*yopT*^{C139A}) or YopT (*yopE*^{R144A}) strains at an MOI of 30 for 90min. In (C,D) cells were further left uninfected or infected with Δ*yopM Yptb* at an MOI of 30 for 90 mins. Unprimed BMDMs or treatment with vehicle DMSO were used as controls. A,C) Immunoblot analysis of BMDM lysates. B,D) Mature IL-1β in supernatants as quantified by ELISA. Each data group is presented as an average (error bars are standard deviation) of technical replicates from one experiment.

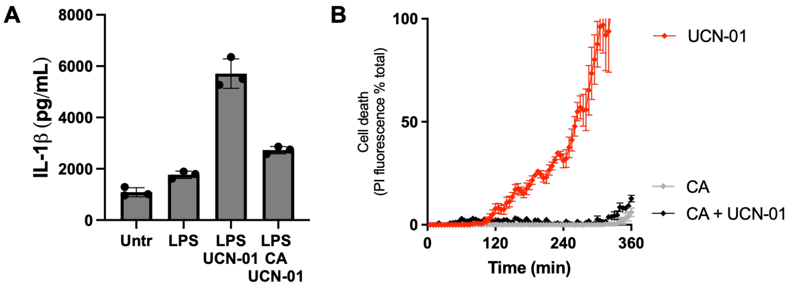

Fig. S3. CA inhibits UCN-01-mediated IL-1β release and cell death in human U937 cells expressing M694V pyrin variant. PMA-differentiated U937 *MEFV*^{KO} cells expressing p.M694V *MEFV* variant were primed with LPS (50ng/ml) for 2.5 hrs and treated with 12.5μM UCN-01 with or without a 30 min pre-treatment with CA (40nM). A) IL-1β level in the supernatant was quantified by ELISA at 3 hrs post-treatment. Untreated cells (Untr) were analyzed in parallel. B) Propidium iodide (PI) influx/fluorescence was monitored every 5 min for 6h. Each data group is presented as an average (error bars are standard deviation) of three technical replicates from one experiment.

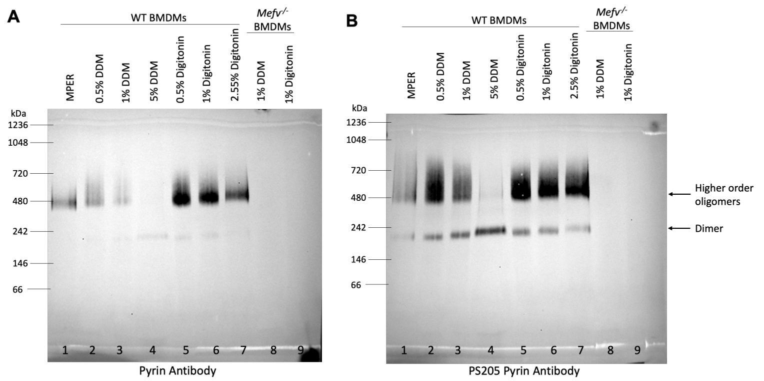

Fig.S4. BMDM lysates prepared with digitonin are optimal for detection of pyrin oligomers using BN-PAGE and immunoblotting. LPS-primed WT and *Mefv*$^{-/-}$ BMDMs were lysed in various non-ionic detergent concentrations as indicated. Lysate samples were separated by BN-PAGE and immunoblotted to visualize total (A) and PS205 pyrin (B) oligomers. Positions of BN-PAGE molecular weight standards are shown on the left of each panel, and dimer and higher order oligomer are indicated on the right of (B).

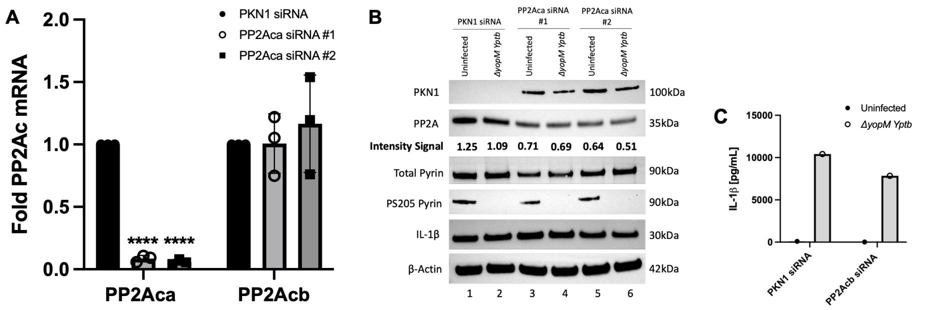

Fig.S5. Individual siRNA knock-down of PP2Aca and PP2Acb in BMDMs does not prevent pyrin inflammasome activation. WT BMDMs were electroporated with siRNAs targeting PKN1 (400pmol), PP2Aca (1000pmol), or PP2Acb (500pmol). 24hr after electroporation the BMDMs were LPS-primed. 48hr following electroporation, cells were either left uninfected or infected with Δ*yopM Yptb* at an MOI of 30 for 90 mins. A) RT-qPCR analysis of mRNA transcripts of PP2Aca and PP2Acb in uninfected BMDMs. Results were normalized to Hprt mRNA levels. Each data group is presented as an average (error bars are standard deviation) of three independent experiments. One-way ANOVA was applied to calculate significance and p-values as compared to PKN1 control siRNA are indicated. P-value<0.05 was considered significant; <0.0001 (****). B) Immunoblot analysis of BMDM lysates. Quantified immunoblot band intensity signals from one experiment representing PP2A/β-Actin are indicated below the respective blot image. C) Mature IL-1β in supernatants as quantified by ELISA from one experiment.
